# Constitutively Active Androgen Receptor Variants Upregulate Expression of Mesenchymal Markers in Prostate Cancer Cells

**DOI:** 10.1371/journal.pone.0063466

**Published:** 2013-05-02

**Authors:** Félicie Cottard, Irène Asmane, Eva Erdmann, Jean-Pierre Bergerat, Jean-Emmanuel Kurtz, Jocelyn Céraline

**Affiliations:** 1 INSERM U1113, Fédération de Médecine Translationnelle de Strasbourg (FMTS), Université de Strasbourg, Strasbourg, France; 2 CHRU Strasbourg, Hematology and Oncology Unit, Strasbourg, France; Innsbruck Medical University, Austria

## Abstract

Androgen receptor (AR) signaling pathway remains the foremost target of novel therapeutics for castration-resistant prostate cancer (CRPC). However, the expression of constitutively active AR variants lacking the carboxy-terminal region in CRPC may lead to therapy inefficacy. These AR variants are supposed to support PCa cell growth in an androgen-depleted environment, but their mode of action still remains unresolved. Moreover, recent studies indicate that constitutively active AR variants are expressed in primary prostate tumors and may contribute to tumor progression. The aim of this study was to investigate the impact of constitutively active AR variants on the expression of tumor progression markers. N-cadherin expression was analyzed in LNCaP cells overexpressing the wild type AR or a constitutively active AR variant by qRT-PCR, Western blot and immunofluorescence. We showed here for the first time that N-cadherin expression was increased in the presence of constitutively active AR variants. These results were confirmed in C4-2B cells overexpressing these AR variants. Although N-cadherin expression is often associated with a downregulation of E-cadherin, this phenomenon was not observed in our model. Nevertheless, in addition to the increased expression of N-cadherin, an upregulation of other mesenchymal markers expression such as *VIMENTIN, SNAIL* and *ZEB1* was observed in the presence of constitutively active variants. In conclusion, our findings highlight novel consequences of constitutively active AR variants on the regulation of mesenchymal markers in prostate cancer.

## Introduction

Prostate cancer (PCa) is the most common cancer in men over 50 years of age and the second cause of male mortality due to cancer in Europe. Androgens signaling plays a key role in PCa cells proliferation or survival [1], and androgen withdrawal remains the main treatment for local recurrence and androgen-dependent metastatic PCa. However, the benefit of this therapy is transient and all tumors ultimately recur as castration-resistant PCa (CRPC).

Genetic and splicing events affecting the androgen receptor (AR) gene have been linked to CRPC. Constitutively active AR variants, lacking the carboxy-terminal region that encompasses the ligand binding domain and the activation function 2, might contribute to the progression of PCa into castration resistance. These constitutively active AR variants result from premature stop codons due to nonsense mutations as reported for the ARQ640X [2], [3], [4], [5] or from alternative splicing with the retention of a cryptic exonic sequence as described for AR-V7 [4], [6], [7], [8], .

The role of constitutively active AR variants in CRPC has been shown in many studies [7], [8], [11], [12]. The expression of these truncated AR variants is increased by a 20-fold in CRPC compared with localized PCa [9], and is correlated with the capacity of PCa cells to grow *in vitro* and *in vivo* in the absence of androgen [7]. However, the exact molecular mechanisms leading to their activation and their mode of action in PCa and CRPC remain unclear.

Recent studies suggest that constitutively active AR variants could play a role in tumor progression. Indeed, although these constitutively active AR variants are already expressed in primary prostate tumors, their expression is all the more expressed in bone metastasis [8]. Furthermore, their expression is associated with an increase of NFAT (Nuclear factor of activated T-cell) and AP-1 (Activator Protein-1) activity, two transcription factors involved in cell proliferation, migration and survival [13].

N-cadherin, which belongs to cadherin superfamily, is located at adherens junctions in nervous, endothelial or mesenchymal cells and is involved in tumor progression [14], [15]. Indeed, N-cadherin expression is increased in most cancers and promotes tumor cells migration, invasion and survival [14]. Increased N-cadherin expression is also associated with epithelial-mesenchymal transition (EMT), a phenomenon characterized by a decrease of epithelial markers such as E-cadherin and an increase of mesenchymal markers such as Vimentin or N-cadherin [16], [17], [18], [19]. These molecular and cellular modifications play an important role in tumor cells dissemination at secondary sites [20], 21.

More recently, studies have shown that castration-resistant PCa is associated with an upregulation of N-cadherin expression in cellular models as well as PCa xenografts and clinical samples of CRPC [22], [23], [24]. Moreover, monoclonal antibodies against N-cadherin have been shown to delay the emergence of castration resistance and to reduce the growth of CRPC xenografts [23]. Taken together, these data show that there is a correlation between N-cadherin expression and resistance to castration. Nevertheless, molecular mechanisms whereby N-cadherin expression is increased in CRPC remain unknown.

The aim of this work was to show a possible link between the presence of constitutively active AR variants and the expression of tumor progression markers. More particularly, we focused on the impact of constitutively active AR variants on the expression of N-cadherin and other mesenchymal markers. In the present study, we have shown that *N-CADHERIN* as well as *VIMENTIN*, *SNAIL* and *ZEB1* are upregulated in the presence of constitutively active AR variants in PCa.

## Materials and Methods

### Cell culture

The human prostate carcinoma LNCaP cell line, clone FGC and the 22Rv1 cell line (ECACC, Salisbury, United Kingdom) was maintained in RPMI-1640 complete medium containing 10% fetal calf serum (FCS), 10 mM HEPES, 2 mM L-glutamine, 100 U/mL penicillin, 100 µg/mL streptomycin (Sigma-Aldrich, France) and 1mM sodium pyruvate (Invitrogen, Fisher Scientific, France).

C4-2B cell line (ViroMed Laboratories, Minnetonka, MN, USA) was maintained in DMEM medium supplemented with 20% Ham's F12, 10% FCS, 100 U/mL penicillin, 100 µg/mL streptomycin, 5 µg/mL insulin, 13.65 pg/mL triiodo-thyronine, 4.4 µg/mL apo-transferrin human, 0.244 µg/mL d-biotin and 12.5 µg/mL adenine (Sigma-Aldrich, France).

### Plasmids and transfection

For immunofluorescence experiments, the wild type androgen receptor (AR) (AR-WT) and the constitutively active AR Q640X and AR Q670X [25] variants were linked to EGFP as previously described [2], [3]. For gene expression analysis and Western-blot, pE-ARWT, pE-ARQ640X and pE-AR-V7 plasmids were constructed by inserting the corresponding AR cDNA between the NheI and BamHI sites in pEGFP-C3.

For transfections, the JetPEI^TM^ transfection reagent (Polyplus Transfection, Ozyme, France) was used according to the manufacturer's protocol. LNCaP cells were seeded in 10 cm dishes at 1×10^6^ cells/dish or in 6-wells plate at 2×10^5^/well. Three days later, the medium was changed and cells were transfected with 10 µg of the indicated plasmid using 20 µl of JetPEI transfection reagent for 10 cm dishes or with 3 µg of plasmid using 6 µl of JetPEI for 6-wells plate. Medium was changed 48 h after and cells were incubated up to 9 days according to the experiments. The medium was changed every two days and for incubations beyond 4 post-transfection days, cells were incubated in the presence of 400 µg/mL geneticin (Invitrogen, France).

### Impact of androgens on N-cadherin expression

LNCaP cells were seeded in 6-wells plate in complete medium and transfected as previously described. Twenty four hours later, medium was changed to phenol red free RPMI-1640 supplemented with 5% dextran-coated charcoal-stripped FCS (DCC-FCS) and with the indicated concentration of dihydrotestosterone (DHT) (Sigma-Aldrich, France) or vehicle (ethanol).

For experiment with MDV3100, transfected LNCaP cells were incubated in RPMI-1640 supplemented with 5% DCC-FCS containing the indicated DHT dose and 100 nM MDV3100 (Enzalutamide, Selleck Chemicals, Euromedex, France) or vehicle (dimethyl sulfoxide, DMSO). To confirm the effects of androgens on N-cadherin expression, 22Rv1 cells were grown in RPMI-1640 with 100 nM or 1 µM MDV3100, or DMSO.

### Cell Sorting

LNCaP cells were seeded in 10cm dishes at 1×10^6^ cells/dish and were transfected with pEGFP-ARWT or pEGFP-ARQ640X. Four days after transfection, cells were trypsinized and sorted thanks to the green fluorescence (EGFP) with a BD FACSAria-II cell sorter (BD Biosciences, Le Pont de Claix, France). Total RNA was extracted from EGFP negative (non-transfected) and EGFP positive (transfected) cells and was used to analyze gene expression by qRT-PCR.

### Quantitative real-time PCR

Total cellular RNA was extracted from cell lines using NucleoSpin® RNA II assay (Macherey-Nagel, France) according to the manufacturer's procedure. RNA concentrations and purity were quantified measuring the absorbance at 260 nm and 280 nm (GeneQuant pro, GE Healthcare, France). The reverse transcription was performed from 400 ng or 1 µg RNA using RT Omniscript assay (Qiagen, Courtaboeuf, France). RNA were diluted into 13 µL and denatured at 65°C during 5 minutes. A 7 µL reaction mix containing 1×RT template, 0.5 mM of each dNTP, 1 µM oligo dT, 10U RNase inhibitor and 4U Omniscript Reverse Transcriptase was added and the reaction was incubated 1 h at 37°C. The reaction was stopped by heating to 93°C for 5 minutes. *N-CADHERIN*, *E-CADHERIN*, *VIMENTIN*, *SNAIL*, *TWIST1*, and *ZEB1* mRNA levels were quantified using real-time PCR with LightCycler 480 (Roche Applied Science, Meylan, France). For PCR reactions, 5 µL LightCycler® 480 SYBR Green I Master (Roche, Molecular Diagnostics, Mannheim, Germany) and 1 µL specific primers (Table 1) (Qiagen, QuantiTect Primers, Courtaboeuf, France) were mixed with 4 µL of 1∶5 cDNA dilution. Results were normalized using housekeeping gene *β-ACTIN* or *PBGD* (Porphobilinogen deaminase) (Qiagen, QuantiTect Primer). Amplification specificity was verified by analyzing melting curve and by electrophoresis migration. All experiments were realized in triplicate and repeated 3 times. Relative quantification was used to determinate fold change in expression level by the ΔΔCt method. Each value is expressed as the mean ΔΔCt ± SEM. Results were analyzed with Student t test and *p*-value <0.05 was considered significant.

**Table 1:** List of primers used in qRT-PCR experiments.

Genbank	QuantiTect reference	Hybridization temperature (°C)	Amplicon length (bp)	Amplified exons
*β-ACTIN (ACTB)*	QT01680476	55/60	104	NA
*E-CADHERIN (CDH1)*	QT00080143	55	84	5/6
*N-CADHERIN (CDH2)*	QT00063196	60	102	14/15
*PBGD (HMBS)*	QT00014462	55	107	7/8/9
*TWIST1*	QT00011956	55	127	1/2
*SNAIL (SNAI1)*	QT00010010	60	131	2/3
*VIMENTIN*	QT00095795	60	94	2/3
*ZEB1*	QT01888446	58	105	2/3/4

### Western Blot

Cells were lysed in buffer containing 10 mM Tris-HCl pH7, 140 mM NaCl, 3 mM MgCl2, 0.5×Igepal, 5 mM DTT, 1× phosphatase inhibitor, and 1× protease inhibitor. Protein concentration for each sample was quantified using BCA Protein Assay (Pierce Biotechnology, Inc., Rockford, IL, USA) according to the manufacturer's procedure. A quantity of 15 µg to 100 µg of total proteins was loaded on 7,5% SDS-PAGE. After migration and transfer to nitrocellulose membrane, membranes were saturated with PBS/0.1%Tween/2%ECL and incubated at 4°C overnight with 0.1 µg/mL mouse monoclonal anti N-cadherin (catalog no. 610920, BD Biosciences, France) or 1 µg/mL mouse monoclonal anti AR (catalog no. 554225, BD Biosciences, France) antibody. β-actin (0.2 µg/mL) (catalog no. sc-47778, Tebu-bio, France) was used as internal control. After washes, immunocomplexes were detected with 0.2 µg/mL HRP-conjugated goat anti mouse (catalog no. sc-2005, Tebu-bio, France), or 0.5 µg/mL rat anti mouse IgG2a secondary antibodies (catalog no. 553391, BD Biosciences, France), and finally revealed by chemiluminescence (Immobilon^TM^ Western, Millipore, Molsheim, France).

### Immunofluorescence Staining

Lab-Tek II chamber slides (2 wells) were coated with LNCaP medium for two hours and 1×10^5^ LNCaP cells/well were seeded. LNCaP cells were transfected with 2 µg of pEGFP-WT, pEGFP-ARQ640X or pEGFP-ARQ670X 3 days later and incubated for 4 days. LNCaP cells were rinsed in PBS and fixed with 2% paraformaldehyde. Cells were blocked and permeabilized by 0.1% Triton/1% Bovine Serum Albumin (BSA)/PBS for 30 min at room temperature. Cultures were incubated with 2.5 µg/mL anti N-cadherin mouse monoclonal antibody (catalog no. 610920, BD Biosciences, France) or isotypic antibody (Sigma-Aldrich, Saint-Quentin Fallavier, France) at 4°C overnight. After washing in PBS, LNCaP cells were incubated with 2 µg/mL Alexa Fluor 568-conjugated goat anti mouse (Invitrogen, Fisher Scientific, France) for 1 h and nuclei were stained with 0.1 µg/mL DAPI solution for 20 min at 30°C. Images were captured with the Leica LAS AF6000 fluorescence microscope using LAS AF software (Leica).

## Results

### Constitutively active androgen receptor variants upregulate N-cadherin expression in prostate cancer cells

**Figure 1:**
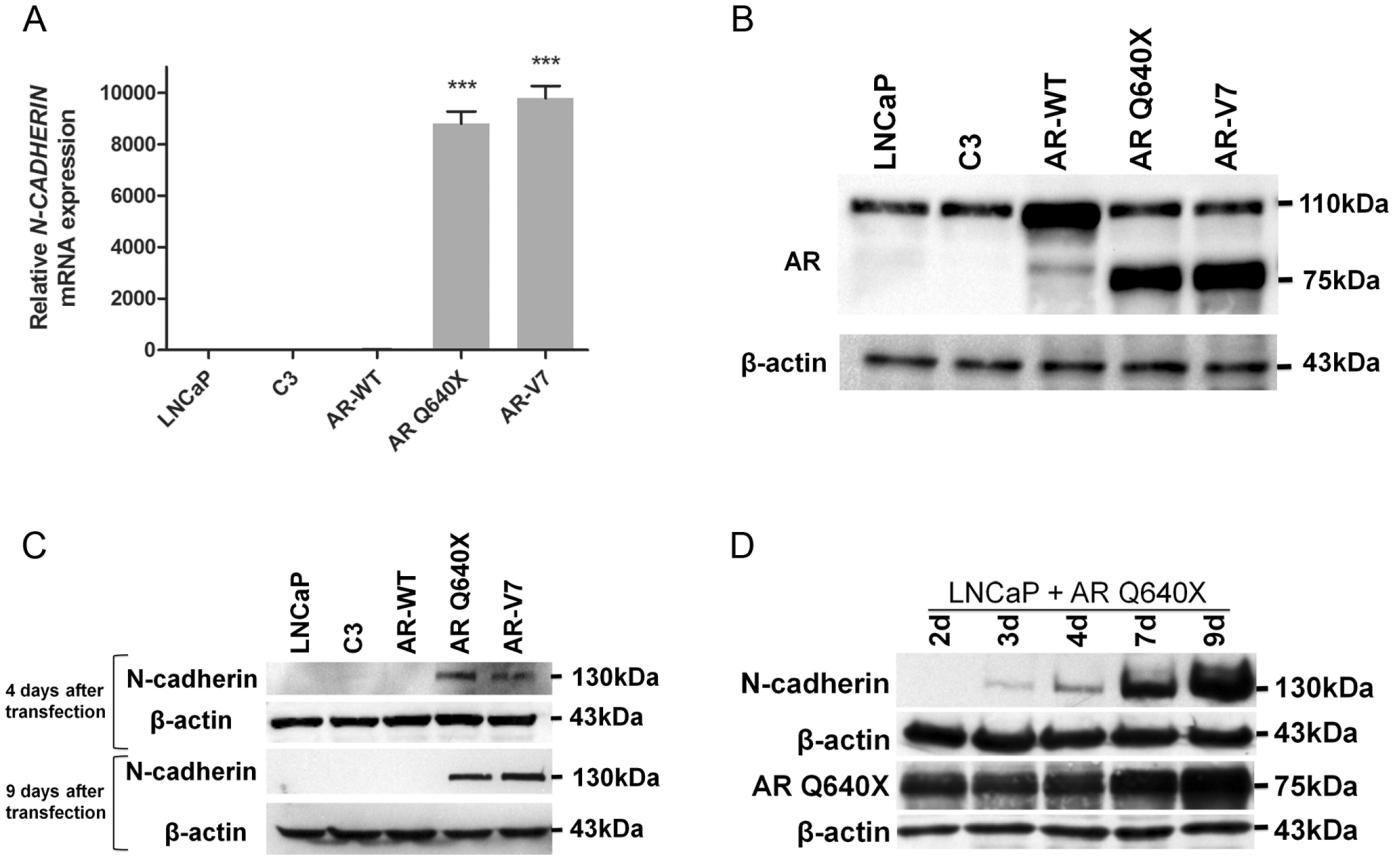
N-cadherin expression is upregulated in the presence of constitutively active androgen receptor variants. A). N-cadherin expression was assessed by qRT-PCR in LNCaP cells overexpressing the constitutively active AR Q640X or AR-V7, or the AR-WT and in cells transfected with the empty plasmid (C3). Cells were grown in complete medium for 9 days after transfection. Parental LNCaP cells were used as control. y-Axis represents the relative fold change compared with control (parental LNCaP cells). *β-ACTIN* was used as the endogenous normalization control. Relative expression is presented as the mean ± SEM from three independent experiments. Each sample is compared one by one by two tail unpaired t test. NS: Not significant * P<0.05, **P<0.01 and ***P<0.001. B). Western Blot showing AR expression in transfected and non transfected LNCaP cells. C). Immunoblot analysis of N-cadherin expression in transfected LNCaP cells 4 and 9 days after transfection. D). Kinetic analysis of N-cadherin expression by Western Blot in LNCaP cells overexpressing AR Q640X from 2 to 9 days after transfection. β-actin was used as loading control.

Constitutively active AR variants have been associated with CRPC. Moreover, some studies showed that CRPC is also associated with an upregulation of N-cadherin expression [22], [23]. We investigated whether constitutively active AR variants upregulate N-cadherin expression in PCa cells. *N-CADHERIN* mRNA level was determined by qRT-PCR in LNCaP cells overexpressing the constitutively active AR Q640X or AR-V7, or the AR-WT as control (Figure 1). *N-CADHERIN* expression remained unchanged in LNCaP cells overexpressing AR-WT compared with controls. Interestingly, *N-CADHERIN* expression was increased by a 8,000-fold in the presence of ARQ640X and AR-V7 (Figure 1A-B). These data were confirmed in C4-2B cells (Figure S1) and at the protein level in LNCaP cells (Figure 1C). In addition, a time course experiment revealed that N-cadherin protein levels were consistently increased from day-3 after LNCaP cells transfection with ARQ640X (Figure 1D).

**Figure 2:**
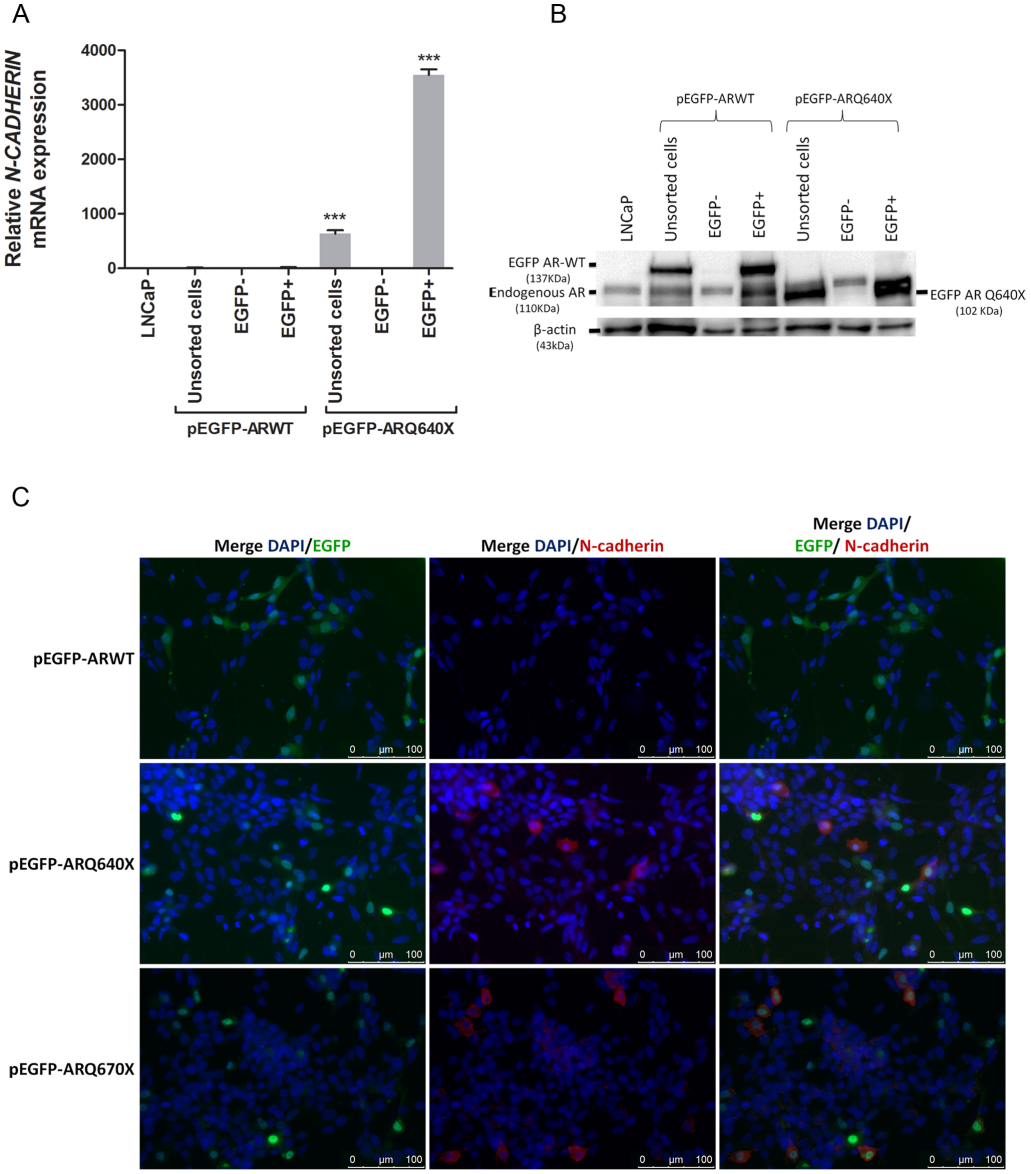
N-cadherin upregulation was restricted to LNCaP cells expressing constitutively active androgen receptor variants. LNCaP cells were transiently transfected with pEGFP-ARWT or pEGFP-ARQ640X plasmid and were sorted 4 days after. A). *N-CADHERIN* expression was analyzed by qRT-PCR in EGFP positive (transfected) and EGFP negative (non-transfected) fractions. B). Androgen receptor (AR) level was analyzed by Western Blot to verify the purity of each fraction after cell sorting. C). Immunofluorescence analysis of N-cadherin (red fluorescence) expression in LNCaP cells transfected with EGFP-tagged (green fluorescence) AR-WT, AR Q640X or AR Q670X expression plasmid. Magnification: ×20.

To confirm these data from transient transfection, a cell-sorting analysis was performed after LNCaP transfection to demonstrate that N-cadherin expression was restricted to cells expressing a constitutively active AR. *N-CADHERIN* expression was analyzed in EGFP negative (non-transfected cells) or EGFP positive (transfected cells) fractions by qRT-PCR. Consistent with above results, *N-CADHERIN* expression was undetectable in both EGFP- negative and positive fractions following LNCaP transfection with pEGFP-ARWT. However, upon transfection with pEGFP-ARQ640X, *N-CADHERIN* expression was increased in EGFP positive cells overexpressing the constitutively active AR, but not in the EGFP negative fraction (Figures 2A–B). These results were further confirmed by immunofluorescence analysis showing an N-cadherin labeling exclusively in EGFP positive cells expressing a constitutively active AR variant (Figure 2C).

Taken together, these data strongly suggest that constitutively active AR variants upregulate N-cadherin expression in PCa cells.

### Androgens negatively regulate N-cadherin expression induced by constitutively active androgen receptor variants

**Figure 3:**
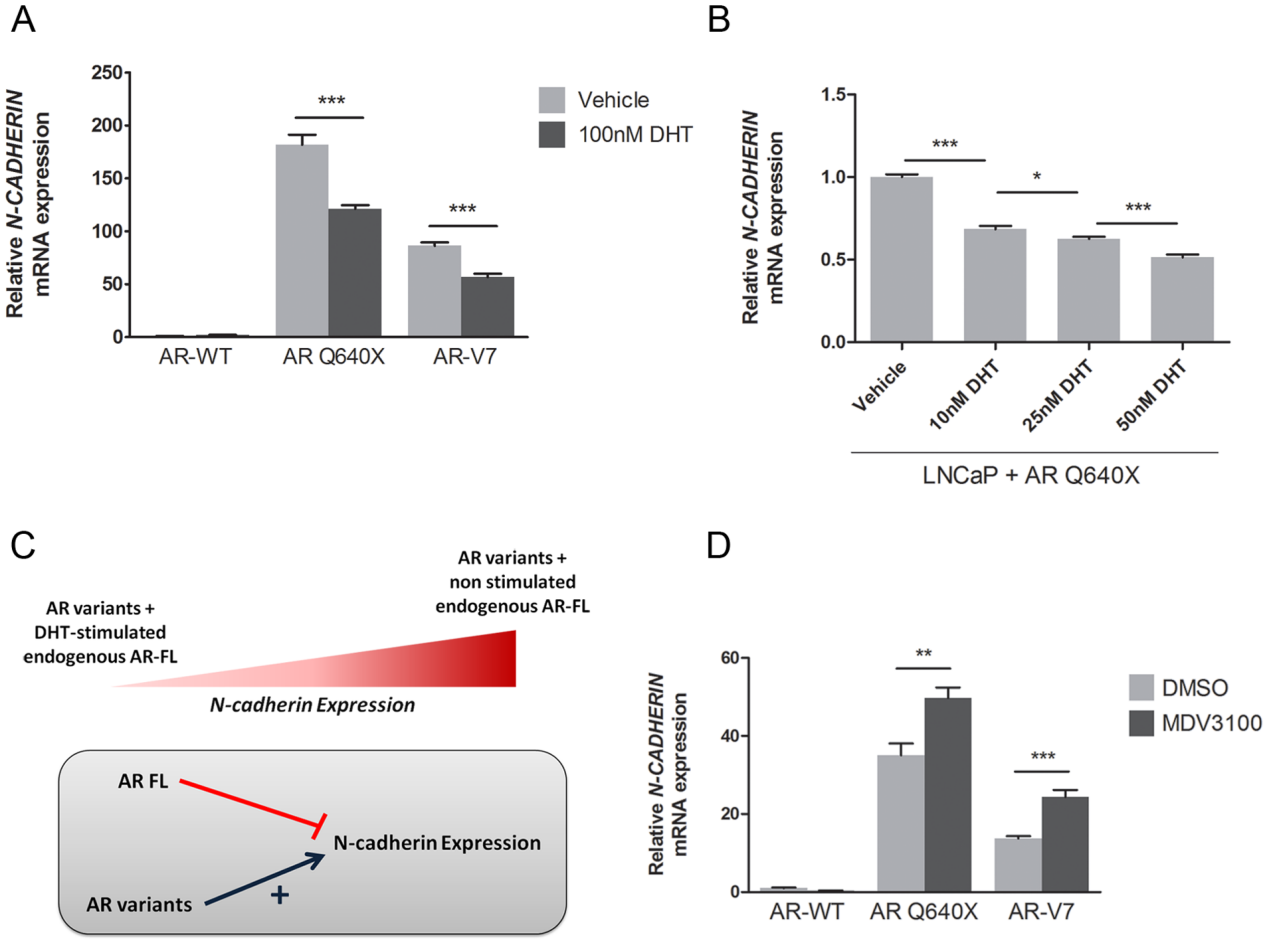
Androgens abrogate N-cadherin upregulation induced by constitutively active androgen receptor variants in LNCaP cells. A). LNCaP cells were grown in RPMI-1640 containing 5% DCC-FCS and 100 nM of DHT or vehicle (EtOH). N-cadherin expression was analyzed by qRT-PCR in LNCaP cells 4 days after transfection with AR-WT or the constitutively active AR Q640X or AR-V7 expression plasmid. B). N-cadherin expression level in LNCaP cells was investigated by qRT-PCR 4 days after transfection with AR Q640X expression plasmid in the presence of different DHT concentrations (10 nM, 25 nM and 50 nM) or vehicle. C). N-cadherin expression induced by constitutively active AR variants (AR variants) was negatively regulated when LNCaP cells were grown in the presence of DHT. We hypothesize that endogenous AR-FL present in LNCaP cells and AR variants could act differently. In this model, DHT-stimulated endogenous AR-FL represses N-cadherin expression whereas AR variants upregulate its expression. D). LNCaP cells overexpressing AR-WT, AR Q640X and AR-V7 were cultured in DCC-FCS medium supplemented with 100 nM of DHT and in the presence of 100 nM of MDV3100 or DMSO as control during 3 days. N-cadherin expression was analyzed by qRT-PCR 4 days after transfection, and was normalized to *β-ACTIN*. The ΔΔCt method was used to calculate relative expression and each value was reported as the mean of ΔΔCt ± SEM. NS: Not Significant * P<0.05, **P<0.01 and ***P<0.001.

A recent study reported that constitutively active AR variants might require a full-length AR (AR-FL) to activate endogenous target genes. To explore the effect of the endogenous AR-FL present in LNCaP cells on the ability of constitutively active AR variants to induce N-cadherin expression, LNCaP cells overexpressing AR-WT or a constitutively androgen variant were incubated in the presence of 100 nM DHT or vehicle, and N-cadherin expression was analyzed by qRT-PCR. In accordance with our previous results, no N-cadherin expression was observed in cells overexpressing AR-WT. Interestingly, a 1.4-fold decrease in N-cadherin expression level was observed when cells overexpressing AR Q640X or AR-V7 were cultured in the presence of 100 nM DHT compared to vehicle (Figure 3A). In addition, this androgen-mediated N-cadherin repression was dose-dependent (Figure 3B). These results suggest that constitutively active androgen receptor variants do not require AR-FL to up-regulate N-cadherin expression. However, DHT-activated AR-FL seems to antagonize effects of constitutively active androgen receptor variants on N-cadherin expression (Figure 3C). To verify this hypothesis, the novel anti-androgen MDV3100 was used to inhibit DHT-activated AR-FL in transfected LNCaP cells. As expected, a further significant increase of N-cadherin expression was observed in LNCaP cells overexpressing AR variants in the presence of 100 nM MDV3100 (Figure 3D). These results were also confirmed in castration-resistant 22Rv1 cells, known to express both AR-FL and constitutively active AR variants. A 2-fold increase in N-cadherin mRNA level was observed when 22Rv1 cells were cultured for 4 days in the presence 100 nM and 1 µM of the anti-androgen MDV3100 (Figure S2).

All together, these data suggest that DHT-activated AR-FL could compete with constitutively active androgen receptor for regulating N-cadherin expression.

### Constitutively active androgen receptor variants are associated with the expression of mesenchymal markers

**Figure 4:**
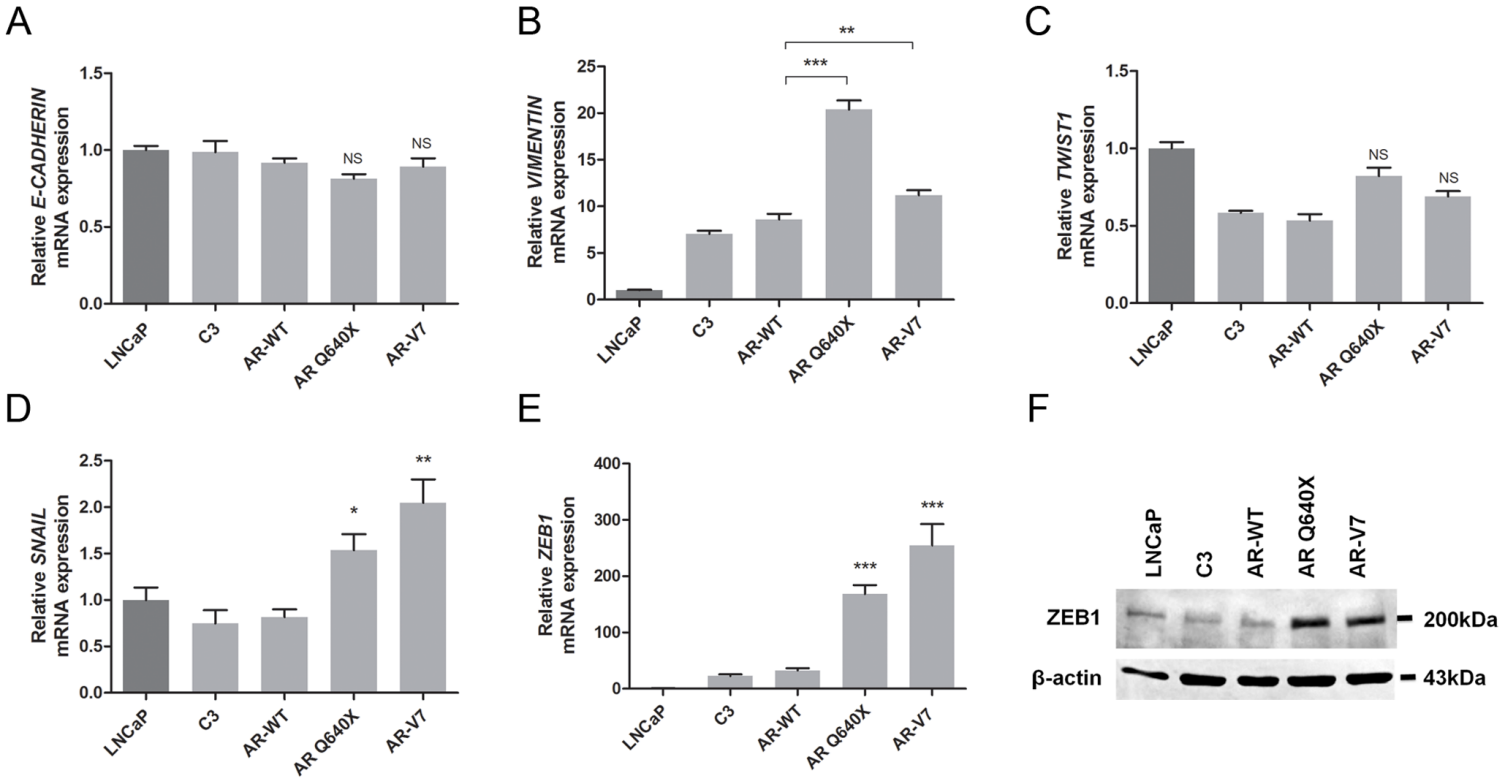
Upregulation of mesenchymal markers by constitutively active androgen variants in prostate cancer cells. LNCaP cells were transfected with the ARWT, ARQ640X or AR-V7 expression plasmid or the empty plasmid (C3). A). *E-CADHERIN*, B). *VIMENTIN*, C). *TWIST1*, D). *SNAIL* and E). *ZEB1* expression levels were analyzed by qRT-PCR at day-9 after transfection. For each sample, expression levels were normalized to *PBGD* or *β-ACTIN* and reported as relative value to LNCaP parental cell line. Values are presented as the mean of ΔΔCt ± SEM. NS: Not Significant * P<0.05, **P<0.01 and ***P<0.001. F). Western Blot showing evolution of ZEB1 expression in LNCaP cells overexpressing constitutively active AR variants. Immunoblot from 100 µg of total protein extracts. β-actin was used as loading control.

It is widely known that in tumor cells, the expression of mesenchymal markers is associated with a down-regulation of epithelial markers. We hypothesized that the upregulation of N-cadherin expression observed in the presence of constitutively active AR variants is accompanied by a decreased expression of E-cadherin. To test this hypothesis, we analyzed E-cadherin expression in LNCaP transfected with ARQ640X, AR-V7 or the wild type AR expression plasmid, or the empty plasmid as control. *E-CADHERIN* mRNA levels in LNCaP cells upon transfection with ARQ640X or AR-V7 expression plasmid did not show any significant difference compared with controls (Figure 4A). These results were further confirmed by Western blot analysis (data not shown), suggesting that the expression of constitutively active AR variants in PCa is associated with a marked increase in N-cadherin expression, but is not correlated with a down-regulation of E-cadherin.

We also investigated whether constitutively active AR expression in PCa cells is associated with other mesenchymal markers. Expression levels of *VIMENTIN* and transcription factors *TWIST1, ZEB1* and *SNAIL* were determined by qRT-PCR at day-9 after LNCaP cells transfection with ARQ640X or AR-V7 expression plasmid, the wild type AR plasmid or the empty vector as controls (Figure 4B–E). *VIMENTIN* expression was increased by a 2.5 and 1.5-fold in LNCaP cells overexpressing ARQ640X and AR-V7 when compared with controls respectively (Figure 4B).

Although Twist1 is known to induce *N-CADHERIN* expression in PCa, no significant difference in the mRNA levels of *TWIST1* was observed (Figure 4C). However, constitutively active AR variants led to a statistically significant increase of *SNAIL* and *ZEB1* mRNA levels (Figure 4D, E). ZEB1 upregulation was also confirmed at the protein level (Figure 4F).

## Discussion

The AR signaling is very important for proliferation and survival of prostate cancer cells. The AR pathway remains activated during the progression of PCa towards a castration-resistant disease and the emergence of constitutively active AR variants lacking the ligand-binding domain is now considered as a major event in CRPC. In spite of some studies suggest that constitutively active AR variants have an impact on tumor progression, their function remains so far unresolved.

In this study, we have shown that N-cadherin is upregulated in LNCaP cells expressing constitutively active AR variants, but not in LNCaP cells overexpressing a full-length AR. These data suggest for the first time that constitutively active AR variants can induce N-cadherin expression. This finding should be connected to recent studies reporting a correlation between CRPC and N-cadherin upregulation [22], [23], [24]. Consistent with these studies, our data suggest that constitutively active AR variants signaling could be a mechanism leading to N-cadherin expression in CRPC.

These findings again highlight the link between AR signaling pathway and N-cadherin expression. Recent studies suggest that AR negatively regulates N-cadherin expression [22], [23]. Indeed, N-cadherin upregulation is associated with a decreased expression of AR in castration-resistant PCa xenografts [23]. Furthermore, the upregulation of N-cadherin observed in the castration-resistant LNCaP-19 cells can be reversed in the presence of androgens [22], [26], [27]. In accordance with these data, we have shown that androgens were associated with a decreased N-cadherin expression in our model overexpressing a constitutively active androgen receptor variant. These results suggest that AR-FL and constitutively active AR variants could act differently (Figure 3C). For example, DHT-stimulated AR-FL might recruit co-repressors and, in turn, represses *N-CADHERIN* expression. Besides, constitutively active AR variants lacking of carboxy-terminal region might behave differently and induce *N-CADHERIN* expression. Furthermore, AR-FL and constitutively active AR variants could compete with each other for regulating N-cadherin expression. Consistent with this hypothesis, *N-CADHERIN* gene contains a cluster of androgen response elements (ARE) repeats in intron 1 [28].

However, constitutively active AR variants could also indirectly control *N-CADHERIN* expression. For example, in prostate cancer, *N-CADHERIN* expression was associated with a nuclear translocation of Twist1 [29]. Although our data showed no significant difference in *TWIST1* mRNA levels, constitutively active AR variants might enhance nuclear translocation of Twist1, which could in turn induce *N-CADHERIN* expression after binding to the E-box within the first intron of *N-CADHERIN*.

These hypotheses deserve to be studied in further studies to understand how constitutively active AR variants regulate *N-CADHERIN* expression.

In this study, we have also shown that constitutively active AR variants were associated with an increased expression of mesenchymal markers as *VIMENTIN*, *SNAIL* and ZEB1. These results are consistent with a recent study, which showed an increase of mesenchymal markers in tumors from patients treated with androgen deprivation therapy [24]. Taken together, our findings suggest that constitutively active AR variants could be associated with EMT process. However, these markers are not consistently associated with EMT. For example, SNAIL confers resistance to apoptosis to tumor cells exposed to ionizing radiations and genotoxic drugs, and enables breast cells to become tumor-initiating cells [30], [31], [32], [33], [34].

The expression of mesenchymal markers reported here in the presence of constitutively active AR variants was not associated with a downregulation of E-cadherin in our model. The inverse correlation between N-cadherin upregulation and E-cadherin downregulation is still debated. Indeed, McKeithen and colleagues, and more recently Tiwari and colleagues report a co-expression of both E- and N-cadherins in tumor cells [35], [36]. In these studies E-cadherin protein displays a different subcellular localization. Moreover, the reported N-cadherin upregulation after castration in LNCaP-19 cells is not accompanied by a decrease of E-cadherin expression in *in vitro* cell culture [22]. Nevertheless, the expected E-cadherin downregulation in this model is only observed in orthotopic tumors after castration, but not in subcutaneous LNCaP-19 tumors, suggesting an important role of the surrounding prostatic environment for E-cadherin downregulation [22]. Besides, an inverse correlation between castration-induced N-cadherin expression and E-cadherin downregulation has been documented in LAPC9 and LuCaP35 subcutaneous xenografts models [23], [24]. However, this cadherins switch has not been reported in two studies focusing on human clinical prostate tumors, from patients with or without androgen deprivation therapy [22], [24].

Further studies are warranted to understand functional consequences of N-cadherin and other mesenchymal markers upregulation in the presence of constitutively active AR variants. N-cadherin expression is widely associated with tumor progression notably owing to its role in migration and invasion. Indeed, N-cadherin favors the migration of cancer cells via cytoskeleton reorganization and lamellipodia formation [14]. It also promotes the migration of cancer cells establishing homophilic interactions with neighboring tissues such as the stromal tissue or endothelium [37], [38]. N-cadherin expression is also associated with survival in prostate cancer cells and melanoma cells. Indeed, N-cadherin expression can activate the phosphatidylinositol 3-kinase (PI3K)/AKT pathway to inactivate pro-apoptotic proteins and to induce an increase of anti-apoptotic proteins as Bcl-2 [39], [40]. Finally, a recent study showed that N-cadherin could mediate angiogenesis by inducing monocyte chemoattractant protein-1 (MCP-1) expression via the PI3K/AKT pathway [41].

There is presently great interest in the mode of action of constitutively active AR variants in CRPC. In this study, we have shown for the first time that constitutively active AR variants induce N-cadherin expression and other mesenchymal markers in PCa. These findings support the hypothesis that these constitutively active AR variants could contribute to systemic dissemination of PCa cells, and reinforce the importance to target these AR variants in PCa.

## Supporting Information

Figure S1 **N-cadherin expression was upregulated in C4-2B cells in the presence of constitutively active AR variants.** N-cadherin expression was assessed by qRT-PCR in C4-2B cells overexpressing AR Q640X variant or transfected with empty plasmid (C3) 4 days after transfection. Parental C4-2B cells were used as control. *N-CADHERIN* expression was normalized to *β-ACTIN* and calculated using the ΔΔCt method. Results are presented as the mean of ΔΔCt ± SEM from three independent experiments. NS: Not Significant * P<0.05, **P<0.01 and ***P<0.001.
(TIF)

Figure S2 **DHT activated AR-FL repressed N-cadherin expression induced by constitutively active AR variants.** 22Rv1 cells were cultured in complete medium supplemented with 100 nM and 1 µM of MDV3100 or DMSO. N-cadherin expression was analyzed by qRT-PCR four days after and was normalized to *PBGD*. The fold change was expressed as relative values to parental cell line 22Rv1 under normal condition. NS: Not Significant * P<0.05, **P<0.01 and ***P<0.001.
(TIF)
