# Supplementary figures and images for: Constitutively Active Androgen Receptor Variants Upregulate Expression of Mesenchymal Markers in Prostate Cancer Cells
Source: PLoS One. 2013 May 2;8(5):e63466. doi: 10.1371/journal.pone.0063466 (PMC3642121; doi:10.1371/journal.pone.0063466)

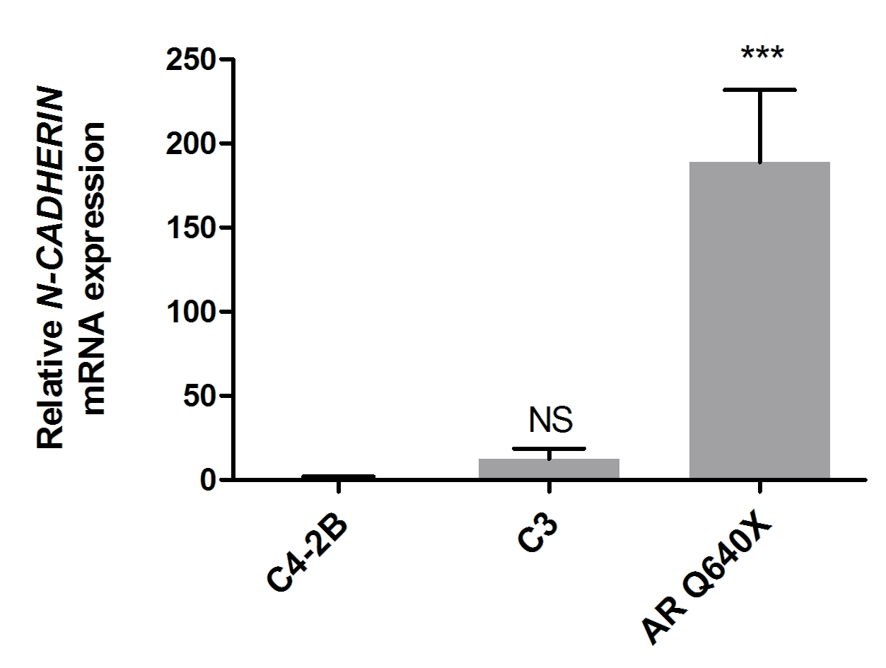

Supplement: Figure S1 — N-cadherin expression was upregulated in C4-2B cells in the presence of constitutively active AR variants. N-cadherin expression was assessed by qRT-PCR in C4-2B cells overexpressing AR Q640X variant or transfected with empty plasmid (C3) 4 days after transfection. Parental C4-2B cells were used as control. N-CADHERIN expression was normalized to β-ACTIN and calculated using the ΔΔCt method. Results are presented as the mean of ΔΔCt ± SEM from three independent experiments. NS: Not Significant * P<0.05, **P<0.01 and ***P<0.001. (TIF) [file pone.0063466.s001.tif]

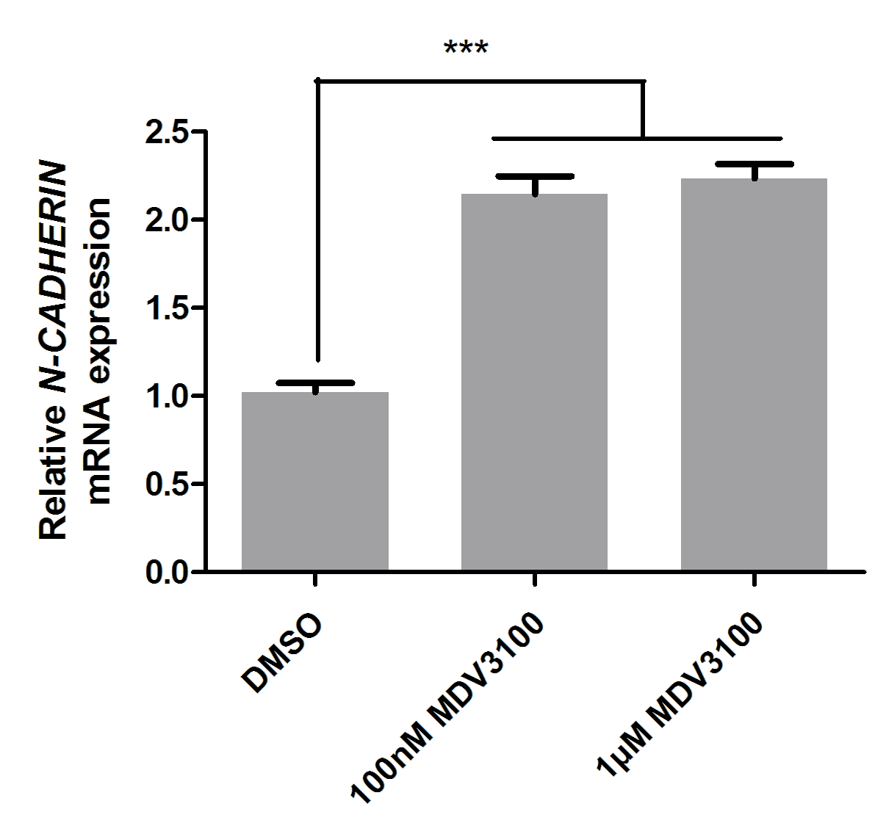

Supplement: Figure S2 — DHT activated AR-FL repressed N-cadherin expression induced by constitutively active AR variants. 22Rv1 cells were cultured in complete medium supplemented with 100 nM and 1 µM of MDV3100 or DMSO. N-cadherin expression was analyzed by qRT-PCR four days after and was normalized to PBGD. The fold change was expressed as relative values to parental cell line 22Rv1 under normal condition. NS: Not Significant * P<0.05, **P<0.01 and ***P<0.001. (TIF) [file pone.0063466.s002.tif]
